# Anti-estrogenic and anti-aromatase activities of citrus peels major compounds in breast cancer

**DOI:** 10.1038/s41598-021-86599-z

**Published:** 2021-03-29

**Authors:** Dina M. El-Kersh, Shahira M. Ezzat, Maha M. Salama, Engy A. Mahrous, Yasmeen M. Attia, Mahmoud Salama Ahmed, Mohey M. Elmazar

**Affiliations:** 1 Department of Pharmacognosy, Faculty of Pharmacy, The British University in Egypt, El Sherouk City, Suez Desert Road, Cairo, 11837 Egypt; 2 Department of Pharmacognosy, Faculty of Pharmacy, Cairo University, Kasr El-Aini Street, Cairo, 11562 Egypt; 3 Department of Pharmacognosy, Faculty of Pharmacy, October University for Modern Sciences and Arts (MSA), 6th October, 12451 Egypt; 4 Department of Pharmacology, Faculty of Pharmacy, The British University in Egypt, El Sherouk City, Suez Desert Road, Cairo, 11837 Egypt; 5 Division of Cardiology, Department of Internal Medicine, The University of Texas Southwestern Medical Center, Dallas, TX USA

**Keywords:** Breast cancer, Medicinal chemistry, Cancer

## Abstract

Estrogen signaling is crucial for breast cancer initiation and progression. Endocrine-based therapies comprising estrogen receptor (ER) modulators and aromatase inhibitors remain the mainstay of treatment. This study aimed at investigating the antitumor potential of the most potent compounds in citrus peels on breast cancer by exploring their anti-estrogenic and anti-aromatase activities. The ethanolic extract of different varieties of citrus peels along with eight isolated flavonoids were screened against estrogen-dependent breast cancer cell lines besides normal cells for evaluating their safety profile. Naringenin, naringin and quercetin demonstrated the lowest IC_50s_ and were therefore selected for further assays. In silico molecular modeling against ER and aromatase was performed for the three compounds. In vivo estrogenic and anti-estrogenic assays confirmed an anti-estrogenic activity for the isolates. Moreover, naringenin, naringin and quercetin demonstrated in vitro inhibitory potential against aromatase enzyme along with anticancer potential in vivo, as evidenced by decreased tumor volumes. Reduction in aromatase levels in solid tumors was also observed in treated groups. Overall, this study suggests an antitumor potential for naringenin, naringin and quercetin isolated from citrus peels in breast cancer via possible modulation of estrogen signaling and aromatase inhibition suggesting their use in pre- and post-menopausal breast cancer patients, respectively.

## Introduction

Citrus fruits are still used in folk medicine for the management of various diseases^1,2^. In cancer, its activity was previously correlated with a potent antioxidant action being enriched with polymethoxyflavones. Moreover, in breast cancer, they were thought to upregulate apoptotic signaling pathways interfering with cancer cell survival^2^. Previous studies also shed light on the possible mechanism by which the major compounds in citrus fruits act as anticancer via inducing cell cycle arrest and interfering with metastasis^3^. They also showed beneficial effects as an adjuvant to conventional chemotherapy, such as doxorubicin, increasing its efficacy while reducing its toxic effects on normal cells^4^. To date, most literature focused on the pharmacological properties of the compounds isolated from the fruit rather than the peel, which is considered as a waste product despite the huge health benefits it possesses.

Breast cancer is classified into hormone receptor-positive, human epidermal growth factor receptor-2 (HER2) overexpressing and triple-negative breast cancer. Hormone receptor-positive breast cancer is known to overly express estrogen and/or progesterone. Eighty percent of all breast cancer cases are estrogen receptor (ER) positive where estrogen signaling stimulates the proliferation of breast cancer cells leading to growth of estrogen-responsive tumors.

In premenopausal women, the ovaries are the principal source of estrogen in the form of estradiol, however, in postmenopausal women, the ovaries no longer produce estrogen which is produced in extragonadal sites instead. Endocrine therapy, the conventional treatment for hormone-responsive breast cancer, involves the use of selective estrogen receptor modulators (SERMs), selective estrogen receptor down regulators (SERDs) and aromatase inhibitors. SERMs are exemplified by tamoxifen and SERDs are exemplified by fulvestrant^5^. Interestingly, the latter was also approved for the treatment of postmenopausal women with advanced breast cancer based on Phase III FALCON trial^6^. Aromatase, a key enzyme in breast carcinogenesis, converts adrenal androgen to estrogen by aromatization^7^. Hence, aromatase inhibitors prevent extragonadal estrogen biosynthesis playing an important role in the management of estrogen-dependent breast cancer, particularly in postmenopausal women^8^. Synthetic aromatase inhibitors such as letrozole and anastrozole have been used as supportive treatments for postmenopausal breast cancer patients^9,10^.

A growing body of evidence suggested that the consumption of certain plants and their constituents (mainly flavonoids) may provide protection against certain types of cancer which led women, especially those at high risk of developing the disease, to use some” herbal” products available in the market as dietary supplements for protection against breast cancer. In most cases, these herbal products are not sufficiently evaluated. Among the most potent natural non-steroidal anti-estrogenic and aromatase inhibitors are flavonoids^11,12^. Aromatase inhibition by flavonoids was investigated in human preadipocytes^13^, in breast cancer cell lines^14^, in transformed yeast cell systems^15^, and in fish ovarian microsomal assays^16,17^.

Citrus fruits, including oranges, lemons, grapefruit and mandarins, are among the most abundant crops in the world with an annual production of over 88 million tons^18^. Almost 33% of the citrus crops are industrially processed for juice production. In some countries like Egypt, oranges account for half the overall fruit production with 3.5 million tons and consumption reaching 70%^19,20^. Citrus peels are also highly rich in active molecular components, flavonoids in particular, either polyhydroxylated, polymethoxylated, or a mixture of both which proved potent against various cancers due to their reported anti-oxidant and anti-inflammatory activities^21^.

Herein, this study suggests that flavonoids isolated from citrus peels exhibit anti-estrogenic and anti-aromatase activities suggesting their potential prophylactic and therapeutic use in both pre- and postmenopausal breast cancer patients. To verify this hypothesis, in vitro screening of the anticancer activity against estrogen-dependent breast cancer cell lines (MCF7 and T47D) was performed for the total extracts and isolates of the peels of the common citrus fruits growing in Egypt. Toxicity was also assessed in vitro on normal cells. In silico molecular modeling for the most active compounds on ER and aromatase were also performed where the degree of superimposition was evaluated starting with 4-Androstene-3-17-dione and 17-beta estradiol as query structures followed by semi-flexible docking against the aromatase and ER binding pockets. Estrogenic and anti-estrogenic in vivo assays were conducted for the isolated compounds. Moreover, in vitro anti-aromatase potential was investigated for the active compounds followed by an in vivo evaluation of the anticancer activity on tumor volume and aromatase levels in solid tumors.

## Material and methods

### General

Sephadex LH 20 (Pharmacia, Stockholm, Sweden) and Diaion HP-20 AG were used for column chromatography (CC). Thin-layer chromatography (TLC) was performed on silica gel GF_254_ precoated plates (Fluka, Steinheim, Germany) using the following solvent systems: S_1_, CH_2_Cl_2_/methanol (90:10), S_2_, CH_2_Cl_2_/methanol (80:20), S_3_, ethyl acetate/methanol/water/formic acid (100:16.5:13.5:2.5). The chromatograms were visualized under UV (at 254 and 366 nm) before and after exposure to ammonia vapour and spraying with AlCl_3._ Dibenzylfluorescein, NaOH, NADPH, glucose 6-phosphate, glucose 6-phosphate dehydrogenase, MgCl_2_, and potassium phosphate (pH 7.4) were obtained from Sigma Chemical Co., (St Louis, MO 63103 USA). Aromatase enzyme (CYP19, BD Biosciences, San Jose, CA). Estradiol benzoate, Folone ampoules, Misr Co. for Pharm. Ind. S.A.E. and standard genistein “Adipogen Life Sciences Inc., Germany”.

### Plant material

The Citrus fruits were collected after permission from Agricultural Research Center, Giza, Egypt allocated in “9, Cairo University Road, Oula, Giza District, Giza Governorate”. Collection of plant material was conducted in compliance with the national guidelines. Ten Citrus samples were obtained:

- *Three varieties* of *Citrus sinensis* L.: navel orange, valencia orange and common baladi orange.
- *Citrus aurantifolia* (Christm.) Swingle (Egyptian Lime).
- *Citrus tangerina* Tanaka (clementine), *Citrus reticulata* Blanco (Ponkan tangarine), *Citrus deliciosa* Tenore. (Mediterranean mandarin).
- *Citrus aurantium* L. (bitter or sour orange).
- *Two varieties* of *Citrus paradisi* Macfad.: grapefruit star ruby red and grapefruit Duncan.

The plants were authenticated by Dr. Reem Samir Hamdy, Lecturer of Plant Taxonomy, Botany Department, Faculty of Science, Cairo University, Giza, Egypt. Voucher samples no (2-2-2014). The plants were deposited at the Museum of the Pharmacognosy Department, Faculty of Pharmacy, Cairo University.

### Extraction and fractionation

The *Citrus* fruits were separated into edible and inedible portions (peels), and the peels were dried in shade and then powdered and kept at 2–4 °C till used. The powdered peels (500 g) of each of the investigated *Citrus* fruits were extracted by 95% ethanol by cold maceration (3 × 3 L) till exhaustion. The combined ethanol extract in each case was evaporated under reduced pressure (at 40 °C) till dryness to yield 131, 150, 130, 100, 180, 105, 83, 120, 127 and 115 g of navel orange, valencia orange, baladi orange, Egyptian lime, *Citrus tangerina*, *Citrus reticulata*, *Citrus deliciosa*, sour orange, grapefruit Duncan and grapefruit star ruby red, respectively. The ethanolic extracts of the investigated peels were subjected to anticancer screening against estrogen-dependent breast cancer cell lines, as will be explained later.

### Purification of the bioactive extracts

The ethanolic extracts of the peels of *C. sinensis* (Valencia), *C. aurantifolia*, *C. tangerina*, *C. aurantium* and *C. paradisi* (Duncan) (100 g of each extract) were suspended separately in distilled water and subjected first to defatting by liquid–liquid extraction using methylene chloride (CH_2_Cl_2_) and then the defatted extract was used for isolation of the major compounds.

### Isolation of the major compounds

The defatted extract from the five bio active *Citrus* species; *C. sinensis* (Valencia), *C. aurantifolia*, *C. tangerina*, *C. aurantium* and *C. paradisi* (Duncan) were purified by loading on diaion HP-20 AG column, elution was carried out with water and methanol, which were monitored by TLC. The promising fraction in each case was purified over several sephadex LH20 columns using water or water–methanol as eluent. Purification of the defatted extract of *C. sinensis* (Valencia) yielded **C1** (100 mg) and **C2** (800 mg), *C. aurantifolia* yielded **C2** (905 mg), **C3** (600 mg), **C4** (530 mg) and **C5** (430 mg), *C. tangerina* yielded **C1** (298 mg), **C6** (200 mg) and **C7** (125 mg), *C. aurantium* produced **C5** (510 mg) and *C. paradisi* (Duncan) yielded **C8** (250 mg). The structures of the eight isolated compounds were investigated according to their physicochemical properties and applying different spectroscopic techniques. Structures of the isolated compounds are shown in (Supp. Fig. S1). The isolated compounds were also subjected to screening against estrogen-dependent breast cancer cell lines, following the methods described in “Assessment of cytotoxic activity” section.

### Assessment of cytotoxic activity

The cytotoxic activity of the ethanolic extracts of the peels along with the isolated major phenolics was tested against two human estrogen-dependent breast cancer cell lines, namely, MCF-7 and T47D, as well as the normal human HFB4 cells for assessment of toxicity, using the sulforhodamine B (SRB) assay^22^. The assessment was performed in the National Cancer Institute in Egypt (NCI). The half maximal inhibitory concentration (IC_50_) values were then calculated from three independent experiments, three replicates each for each sample.

Since quercetin (C2), naringenin (C4) and naringin (C8) showed the highest cytotoxicity against estrogen-dependent breast cancer cells meanwhile demonstrated favorable safety profiles on HFB4 cells, they were enrolled in further in-silico and biological anti-estrogenic and anti-aromatase assays.

### In silico molecular modeling for the most potent isolated compounds against ER and aromatase enzyme

The two-dimensional (2D) chemical structures of quercetin, naringin, and naringenin were exported to three-dimensional (3D) structures to be energy minimized using MMFF94 force field. Crystal structures of aromatase enzyme (PDB ID: 3EQM) and ER ligand binding domain (PDB ID: 1ERE) were downloaded to be prepared in OpenEye molecular modeling software environment.

#### Degree of superimposition evaluation

All the energy minimized structures underwent using virtual Rapid Overlay Chemical Structures (vROCS) via assessment of Tanimoto scores starting with 4-Androstene-3-17-dione and 17-beta estradiol as query structures.

#### Docking and scoring assessment

The energy minimized compounds underwent multi-conformer generation using Omega, followed by semi-flexible docking using FRED against the aromatase binding pocket (PDB ID: 3EQM) and ER binding pocket (PDB ID: 1ERE). Visualization was conducted using Vida visualizer^23–26^.

### In vivo evaluation of estrogenic and antiestrogenic activity for the most potent isolated compounds

#### Animals

Immature female Swiss albino mice (8–13 g) were obtained from the animal house of the Faculty of Pharmacy, The British University in Egypt. All experimental procedures were conducted in compliance with the *Animal Research: Reporting of In Vivo Experiments* (ARRIVE) guidelines and the National Institutes of Health guide for the care and use of Laboratory animals (NIH Publication No. 85-23, revised 2011) and were approved by the Ethics Committee, Faculty of Pharmacy, The British University in Egypt (Ex-2101). All experiments were performed during the light phase of a light/dark cycle that was started 1 week after acclimatization. The room temperature was adjusted at 25 ± 2 °C. Mice had free access to food and water during the experiments.

#### Experimental design and groups

The experimental design was adopted from^27,28^. The mice were randomly divided into 9 groups (7–8 mice each) as follows: Group 1: Negative control (NC) receiving only olive oil, s.c.; Group 2: Estradiol positive control (Est.) dissolved in olive oil given s.c. at a dose of 12.5 µg/kg. *For estrogenic activity*, Group 3: Genistein phytoestrogen positive control (Gein.) treated group; Group 4: Quercetin (Qrt.) treated group; Group 5: Naringenin (Narn.) treated group and Group 6: Naringin (Nar.) treated group. *For anti-estrogenic activity*, estradiol was given after treatments by 6 h for Groups 7, 8 and 9 representing Qrt., Narn. and Nar., respectively. All treatments including genistein were given at a dose of 30 mg/kg dissolved in Tween 80 (1%), i.p., for 7 days. The animals’ weights were recorded daily to ensure the safety of the doses used. On the 7th day, the animals were sacrificed, the morphological appearance of the livers was observed, and the uteri were also isolated and weighed.

#### Experimental parameters

The in vivo estrogenic and anti-estrogenic activities were assessed. The vaginal opening on each day of the study was checked as a sign of puberty. On the 7th day, vaginal swab was taken from the opened vagina to examine the cornification level under light microscope (4× lens). The uteri were excised and weighed after sacrification of mice under anesthesia.

### Evaluation of anti-aromatase activity for the most potent isolated compounds

#### In vitro aromatase inhibition assay

In vitro aromatase inhibition assay was performed for quercetin, naringenin and naringin. Aromatase inhibition was quantified by measuring the fluorescent intensity of fluorescein, the hydrolysis product of dibenzylfluorescein, by aromatase, as previously described^29^. In brief, the test substance (10 µL) was pre-incubated with the NADPH regenerating system (90 µL of 2.6 mM NADP^+^, 7.6 mM glucose 6-phosphate, 0.8 U/mL glucose 6-phosphate dehydrogenase, 13.9 mM MgCl_2_, and 1 mg/mL albumin in 50 mM potassium phosphate, pH 7.4) for 10 min at 37 °C before 100 µL of the enzyme and substrate mixture [4 pmol/well enzyme (CYP19, BD Biosciences, San Jose, CA), 0.4 µM dibenzylfluorescein, and 4 mg/mL albumin in 50 mM potassium phosphate, pH 7.4] were added. Then, the reaction mixture was incubated for 30 min at 37 °C to allow aromatase to generate the product and quenched with 75 µL of 2 N NaOH. After the reaction was terminated, shaking was done for 5 min followed by incubation for 2 h at 37 °C to enhance the noise/background ratio, then fluorescence was measured at 485 nm (excitation) and 530 nm (emission). Three independent experiments were performed in duplicates, and the average values were used to construct the dose–response curves. At least four concentrations of each test substance were used, and the IC_50_ values were calculated and compared to ketoconazole as reference standard at a concentration of 0.1 µg/mL.

#### In vivo evaluation of anticancer potential and aromatase levels in solid tumors

##### Ehrlich ascites carcinoma (EAC) solid tumor animal model

In order to evaluate the anticancer effect of the active isolates (quercetin, naringenin and naringin) and their potential to decrease aromatase levels in tumours, an animal model of Ehrlich ascites carcinoma (EAC) solid tumour was used followed by aromatase ELISA assay as will be described later. Swiss albino female mice (20–25 g) were used for the experiment. EAC cells were collected from the ascitic fluid of a female Swiss albino mouse bearing a 10-day old ascitic tumor where approximately 2.5 × 10^6^ cells were transplanted, i.m. in the left thigh of female mice. When tumors became palpable (approximately 200–300 mm^3^ in volume), treatment was initiated. Forty EAC-tumour bearing mice were randomly allocated into the following groups (n = 10): (1) PC group: positive control group that received only drug vehicle, (2) Qrt.-treated group: Mice were treated with a daily dose of 100 mg/kg of quercetin^30^ i.p., (3) Narn.-treated group: Mice were treated with a daily dose of 50 mg/kg of naringenin, i.p.^31^, and (4) Nar.-treated group: Mice were treated with a daily dose of 100 mg/kg of naringin, i.p.^32^. Treatment doses were given daily excluding Fridays and Saturdays. Tween 80 (1%) was used as a vehicle. Mice were sacrificed under brief anaesthesia at the 20th day after starting treatment and tumors were collected for further analysis.

##### Tumor volume

Tumor volume was calculated using a digital caliper at days 0, 4, 7, 10, 13, 16, and 19 after treatment, according to the following equation^33^:

$${\text{Tumor volume}} = {\text{Length }}\left( {{\text{mm}}} \right) \, \times \, \left[ {{\text{Height }}\left( {{\text{mm}}} \right)} \right]^{{2}} \times \, 0.{52}.$$

##### Determination of aromatase protein levels in tumors

Excised tumors were homogenized as 10% (w/v) in phosphate buffered saline. Aromatase concentration was then determined in tissue homogenates using Mouse Aromatase ELISA kit (MyBiosource, USA), according to the manufacturer’s instructions. Optical density equivalent to aromatase levels in samples was determined at 450 nm using a microplate reader.

### Statistical analysis

Statistical analysis was performed using GraphPad Prism, version 5.0 (GraphPad Software, CA, USA). Statistical significance was determined at *p* < 0.05 using Chi-square for vaginal opening assay and One-Way ANOVA followed by Dunnett’s multiple Comparisons test for estrogenic activity and Tukey’s multiple comparisons for tumor volume and aromatase concentrations in solid tumors.

## Results

### Identification of the major compounds

Eight compounds were isolated from the five active extracts. The structures of the isolated compounds were investigated using UV, 1D & 2D-^1^H and ^13^C NMR spectroscopy. A flavone; nobiletin (5,6,7,8,3′,4′-hexamethoxy flavone) (**C1**) and a flavonol aglycone quercetin (5,7,3′,4′-tetrahydroxy flavonol) (**C2**) were isolated from Valencia orange, while, *C. aurantifolia* yielded quercetin (**C2**), a flavone glycoside; diosmin (5,3′-dihydroxy-4′-methoxy flavone-7*O*-α-l-rhamnopyranosyl (1 → 6)β-d-glucopyranoside) (**C3)**, two flavanones; naringenin (5,7,4′-trihydroxy flavanone) (**C4**) and hesperidin (5,3′-dihydroxy-4′-methoxy flavanone-7*O*-α-l-rhamnopyranosyl (1 → 6)β-d-glucopyranoside) (**C5**). On the other hand, *C. tangerina* yielded two aglycones; a flavone; nobiletin (**C1**) and a flavanone; hesperitin (5,7,3′-trihydroxy-4′-methoxy flavanone) (**C6)** in addition to a flavonol glycoside; rutin (5,7,3′,4′-tetrahydroxy flavon-3*O*-α-l-rhamnopyranosyl (1 → 6)β-d-glucopyranoside) (**C7**), while *C. aurantium* and *C. paradisi* each yielded a flavonone glycoside; hesperidin (**C5)** and naringin (5,4′-dihydroxy-7*O*-α-l-rhamnopyranosyl (1 → 6) β-d-glucopyranoside) (**C8**), respectively. Chemical structures are shown in (Supp. Fig. S1). The ^1^H and ^13^C NMR data are presented in (Tables 1, 2).

**Table 1:** ^1^H NMR chemical shifts (δ ppm) for compounds **C1–C8** (DMSO-d_6_, 400 MHz).

H	C1	C2	C3	C4	C5	C6	C7	C8
2	–	–	–	5.47 dd (12.6, 2.7)	5.54 dd (12.6, 2.7)	5.46 dd (12.6, 2.7)	–	5.51 dd (12.6, 2.7)
3	6.58 s	**–**	6.78 s	3ax 2.71,dd (17.3) 3eq 3.3, dd, (17.3)	3ax 2.79,dd (17.3) 3eq 3.31, dd, (17.3)	3ax 2.74,dd (17.3) 3eq 3.23, dd, (17.3)	–	3ax 2.73,dd (17,3) 3eq 3.40, dd, (17.3)
6	–	6.20 d (2.1)	6.42 d (2.1)	5.91 s	6.16 d (2.1)	5.91 d (2.1)	6.21 d (2.1)	6.08 d (2.1)
8	–	6.42 d (2.1)	6.72 d (2.1)	5.91 s	6.18 d (2.1)	5.92 d (2.1)	6.40 d (2.1)	6.11 d (2.1)
2′	7.93 d (2.1)	7.69 d (2.1)	7.45 d (2.1)	7.35 d (8.4)	6.97 d (2.1)	6.95 d (2.1)	7.55 d (2.1)	7.34 d (8.4)
3′	–	–	6.92 d (8.4)	6.82 d (8.4)	–	–	–	6.81 d (8.4)
5′	6.96 d (8.4)	6.90 d (8.4)	7.14 d (8.4)	6.82 d (8.4)	6.83 d (8.4)	6.90 d (8.4)	6.86 d (8.4)	6.81 d (8.4)
6`	7.53 dd (2.1,8.4)	7.56 dd (2.1,8.4)	7.58 dd (2.1,8.4)	7.35 d (8.4)	6.99 dd (2.1,8.4)	6.97 dd (2.1,8.4)	7.56 dd (2.1,8.4)	7.34 d (8.4)
Glc-1′′	–	–	5.08 d (7.5)	–	5.01 d (7.5)	–	5.35 d (7.5)	5.16 d (7.5)
Rha-1′′′	–	–	4.55 d (2.1)	–	4.55 d (2.1)	–	5.12 d (2.1)	5.11 d (2.1)
Me-6′′′	–	–	1.08 d (6.4)	–	1.12 d (6.6)	–	1.02 d (6.1)	1.14 d (6.1)
OCH_3_-5	4.09 s	–	–	–	–	–	–	–
OCH_3_-6	4.01 s	–	–	–	–	–	–	–
OCH_3_-7	3.95 s	–	–	–	–	–	–	–
OCH_3_-4`	3.93 s	–	3.87 s	–	3.82 s	3.81 s	–	–
OCH_3_-8,3`	3.93 s	–	–	–	–	–	–	–

Coupling constants are (J in Hz) in parenthesis.

**Table 2:** ^13^C NMR chemical shifts (δ ppm) for compounds **C1**-**C8** (DMSO, 100 MHz).

C	C1	C2	C3	C4	C5	C6	C7	C8
2	161.0	147.8	164.2	79.1	78.6	78.8	158.4	79.2
3	106.7	136.4	103.8	42.7	42.0	41.9	135.5	42.2
4	182.0	176.5	181.9	196.4	197.2	196.2	179.3	197.9
5	144.0	157.1	161.1	164.2	163.2	163.7	162.4	163.4
6	137.9	98.6	99.9	96.0	96.7	96.1	99.8	96.7
7	151.4	164.7	162.9	166.0	165.5	165.3	166.1	165.3
8	148.3	94.1	94.8	95.0	95.4	95.2	94.6	95.5
9	147.6	161.7	156.9	163.6	162.7	162.2	159.3	163.1
10	114.7	103.8	105.9	102.3	103.5	102.7	105.5	103.7
1′	123.9	122.7	122.8	129.9	131.1	130.9	123.0	129.7
2′	108.5	116.2	113.1	128.2	114.3	113.8	117.5	129.0
3′	149.2	146.0	146.7	115.3	146.6	145.9	145.7	116.4
4′	151.9	148.6	151.3	157.9	148.1	148.3	149.6	158.6
5′	111.2	115.6	112.2	115.3	112.2	112.3	116.0	116.4
6′	119.6	121.0	118.9	128.2	118.1	118.4	123.4	129.0
1′′			99.6		99.6		101.5	100.8
2′′			73.0		72.2		74.8	76.6
3′′			76.2		76.5		77.1	77.5
4′′			70.7		70.9		72.3	70.0
5′′			75.6		75.7		76.6	77.5
6′′			66.0		66.2		67.5	60.8
1′′′			100.4		100.8		102.3	97.8
2′′′			70.2		69.7		70.7	70.9
3′′′			69.5		70.5		71.2	70.8
4′′′			72.0		72.0		72.8	72.2
5′′′			68.2		68.5		69.1	68.7
6′′′			17.7		18.1		18.3	18.5
OCH_3_-4′	55.9		56.6		55.8	55.6		
OCH_3_-3′	56.0							
OCH_3_-7	61.6							
OCH_3_-6	61.7							
OCH_3_-8	61.9							
OCH_3_-5	62.2							

### Assessment of cytotoxic activity against estrogen-dependent breast cancer cell lines

As shown in Table 3, the cytotoxic activity of the five active ethanolic extracts along with the isolated flavonoids was investigated against two estrogen-dependent breast cancer cell lines (MCF7 and T47D). The five tested extracts showed significant anticancer activity against both cell lines. The extracts also showed selectivity, as evidenced by their relatively higher IC_50_ values on HFB4 cells. On the other hand, the isolated compounds, namely, quercetin (**C2**), naringenin (**C4**) and naringin (**C8**) showed the highest antiproliferative activities with no remarkable toxicity to HFB4 normal melanocytes within the tested concentration (0–50 µg/mL).

**Table 3:** In vitro anticancer and anti-aromatase activities of citrus peel extracts and the isolated compounds.

The tested samples	IC_50_ values (µg/mL) ± SD			
	Cytotoxic effect on MCF7	Cytotoxic effect on T47D	Cytotoxic effect on HFB4	Aromatase inhibition
Valencia orange (sefi)	12.1 ± 2.30	24.4 ± 3.27	197.0 ± 5.23	ND
Common Baladi orange	ND	ND	ND	ND
Navel orange (sorra)	ND	ND	ND	ND
Bitter orange	12.5 ± 2.77	17.9 ± 3.11	295.0 ± 22.15	ND
Mandarin	ND	ND	ND	ND
Ponkan tangarine	ND	ND	ND	ND
Dancy tangerine	14.5 ± 1.87	12.5 ± 2.26	200.0 ± 6.77	ND
Egyptian lime	5.5 ± 0.49	7.9 ± 1.22	62.0 ± 9.83	ND
Grapefruit Duncan	4.9 ± 0.93	5.9 ± 0.89	64.0 ± 8.66	ND
Grapefruit star ruby red	ND	ND	ND	ND
Standard Ketoconazole	ND	ND	ND	2.0 ± 0.15
C1	–	–	–	ND
C2	2.11 ± 0.71	1.81 ± 0.28	–	0.23 ± 0.01
C3	–	–	–	ND
C4	1.12 ± 0.09	1.93 ± 0.07	–	1.615 ± 0.05
C5	–	–	–	ND
C6	4.96 ± 0.89	3.53 ± 0.19	–	ND
C7	–	–	–	ND
C8	2.00 ± 0.98	3.63 ± 0.10	–	1.894 ± 0.07

*ND* not done.

(−) no activity in a concentration up to 50 µg/mL.

Since quercetin, naringenin and naringin exhibited the highest cytotoxic potential among extracts and other isolated flavonoids while demonstrating safety profiles (on HFB4 cells), they were enrolled in further *in-silico* and biological anti-estrogenic and anti-aromatase assays.

### Docking studies for ER and aromatase enzyme

#### Degree of superimposition assessment

**Figure 1:**
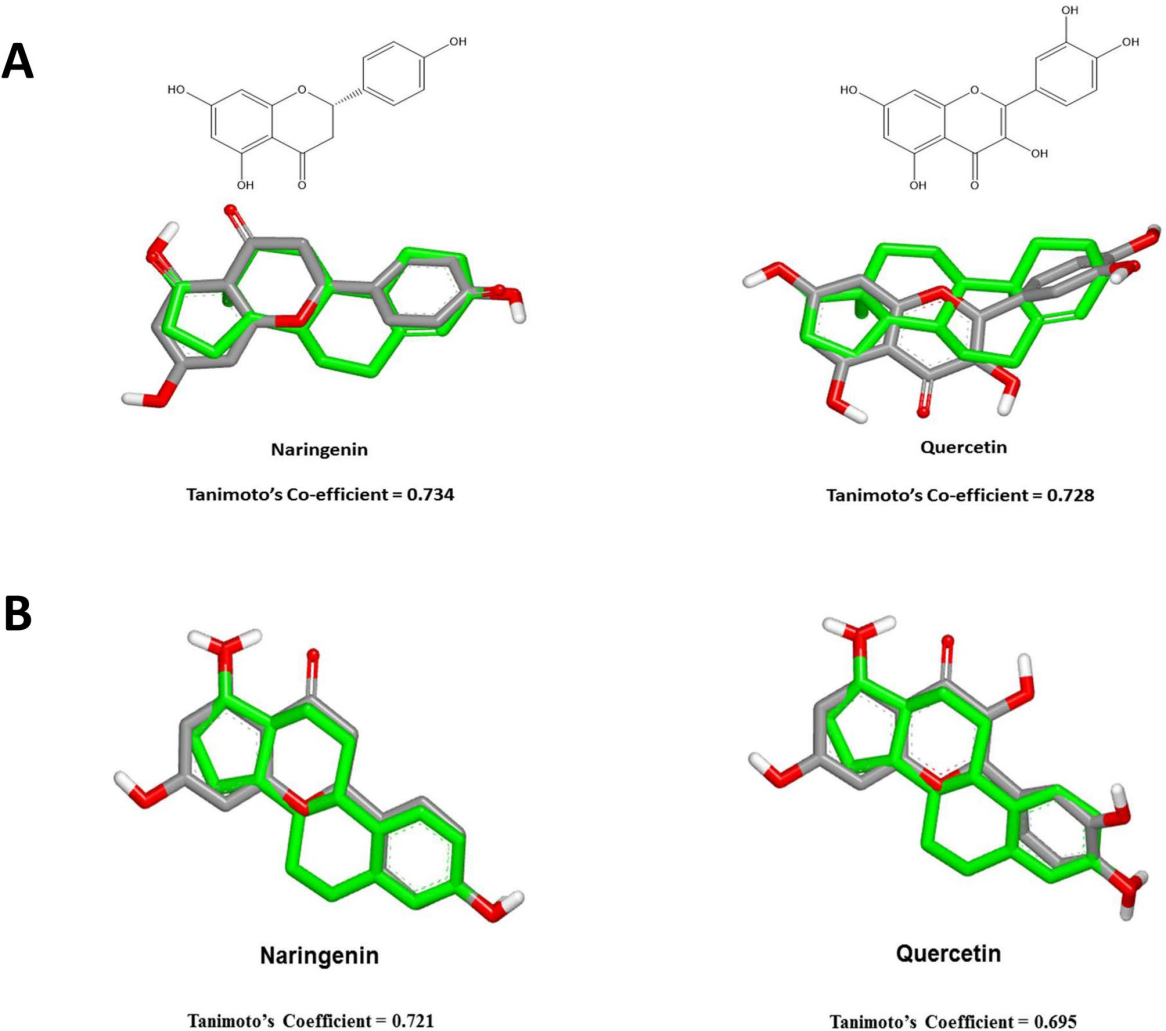
Superimposition of naringenin and quercetin in correlation to (**A**) androsterone and (**B**) 17-beta estradiol, showing Tanimoto co-efficients.

Androsterone structure was used as a starting point for validating the mimicry of the proposed compounds to the aromatase inhibitors via evaluation of the superimposition of the 3D structures. This was done by assessment of shape Tanimoto co-efficient; where naringenin and quercetin showed high degree of superimposition of 0.734 and 0.728, respectively, as shown in Fig. 1A. 17 Beta estradiol structure was used for evaluating the molecular similarity along with the proposed ER targeting ligands; where naringenin and quercetin showed high degree of superimposition of 0.721 and 0.695, respectively, as shown in Fig. 1B.

#### Docking and scoring assessment

Further validation was conducted via receptor-based approach by conducting semi-flexible docking for multi-conformers of naringenin, quercetin and naringin, in comparison with androsterone, against the crystal structure of aromatase enzyme. Naringenin and quercetin managed to bind to the hydrophobic pocket of the catalytic binding domain of the aromatase enzyme, however, they showed different binding behavior via hydrophobic interactions to other neighboring amino acid residues and hydrogen bond interactions.

**Figure 2:**
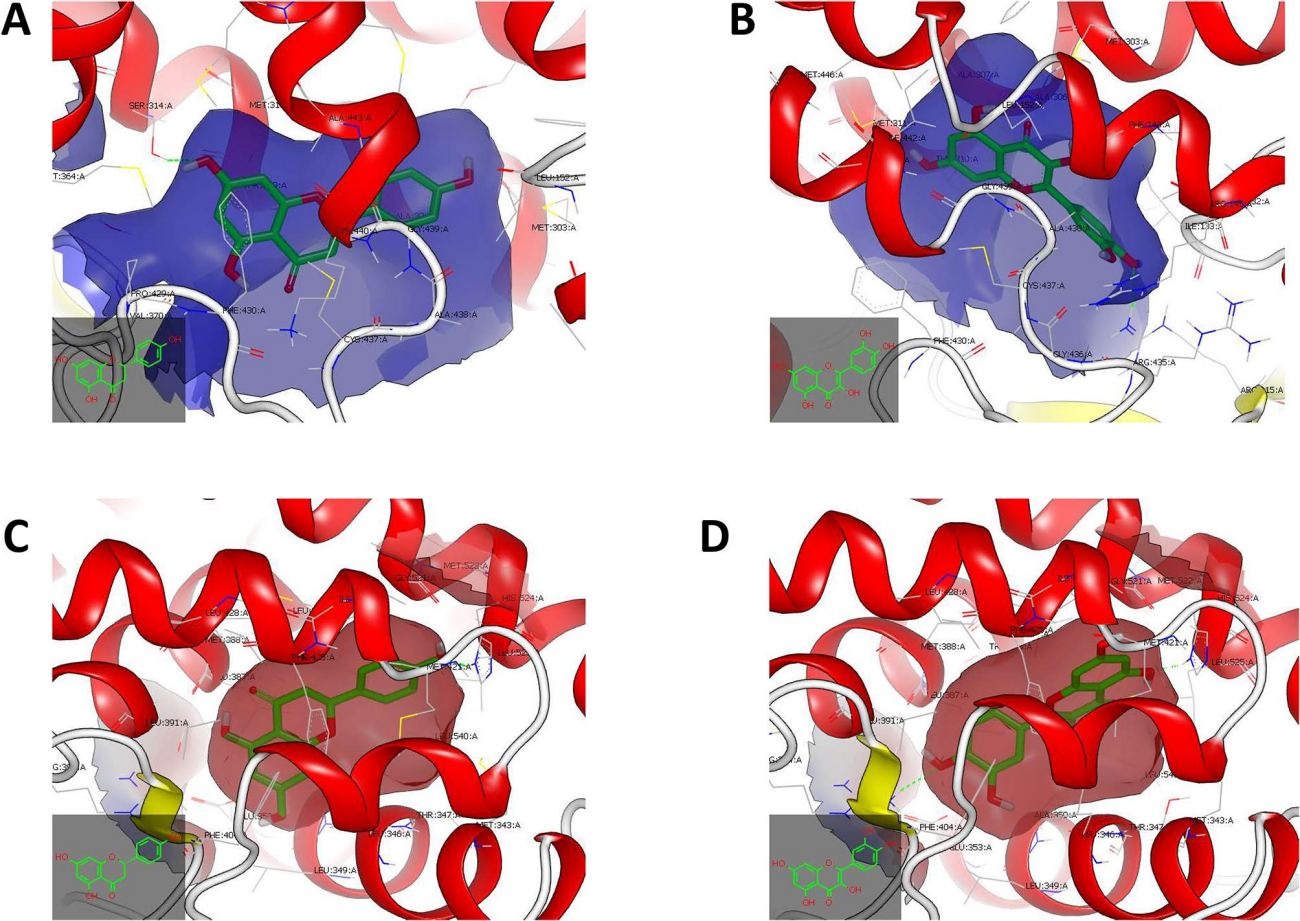
Visual representations of naringenin and quercetin towards aromatase and estrogen receptor ligand binding domains. (**A**) Visual representation of naringenin towards catalytic binding domain of aromatase enzyme, where the dotted green lines refer to the hydrogen bond towards SER 314:A. (**B**) Visual representation of quercetin towards catalytic binding domain of aromatase enzyme, where the dotted green lines refer to the hydrogen bond towards ARG115:A. (**C**) Visual representation of Naringenin towards estrogen receptor ligand binding domain, where the dotted green lines refer to the hydrogen bond towards HIS 524:A. (**D**) Visual representation of Quercetin towards estrogen receptor ligand binding domain, where the dotted green lines refer to the hydrogen bond towards ARG 394:A and HIS 524:A. Visualization was conducted using Vida application from the openeye package (www.eyesopen.com).

Naringenin exhibited hydrophobic–hydrophobic interactions along with aromatase enzyme, showing one hydrogen bonding between –OH of Ring A and Serine 314:A, as shown in Fig. 2A; while quercetin showed one hydrogen bond between –OH group of Ring C and ARG 115:A, as shown in Fig. 2B.

Also, multi-conformers of naringenin, quercetin and naringin, in comparison with 17 beta estradiol, against the crystal structure of ligand binding domain of ER, naringenin and quercetin managed to bind to the hydrophobic pocket of the ligand binding domain of ER, however they showed different binding behavior via hydrophobic interactions to other neighboring amino acid residues and hydrogen bond interactions.

Naringenin exhibited hydrophobic–hydrophobic interactions along with ER along with HIS 524:A, as shown in Fig. 2C; while quercetin showed two hydrogen bonds towards ARG 394:A and HIS 524:A, as shown in Fig. 2D.

### In vivo estrogenic activity assay

**Figure 3:**
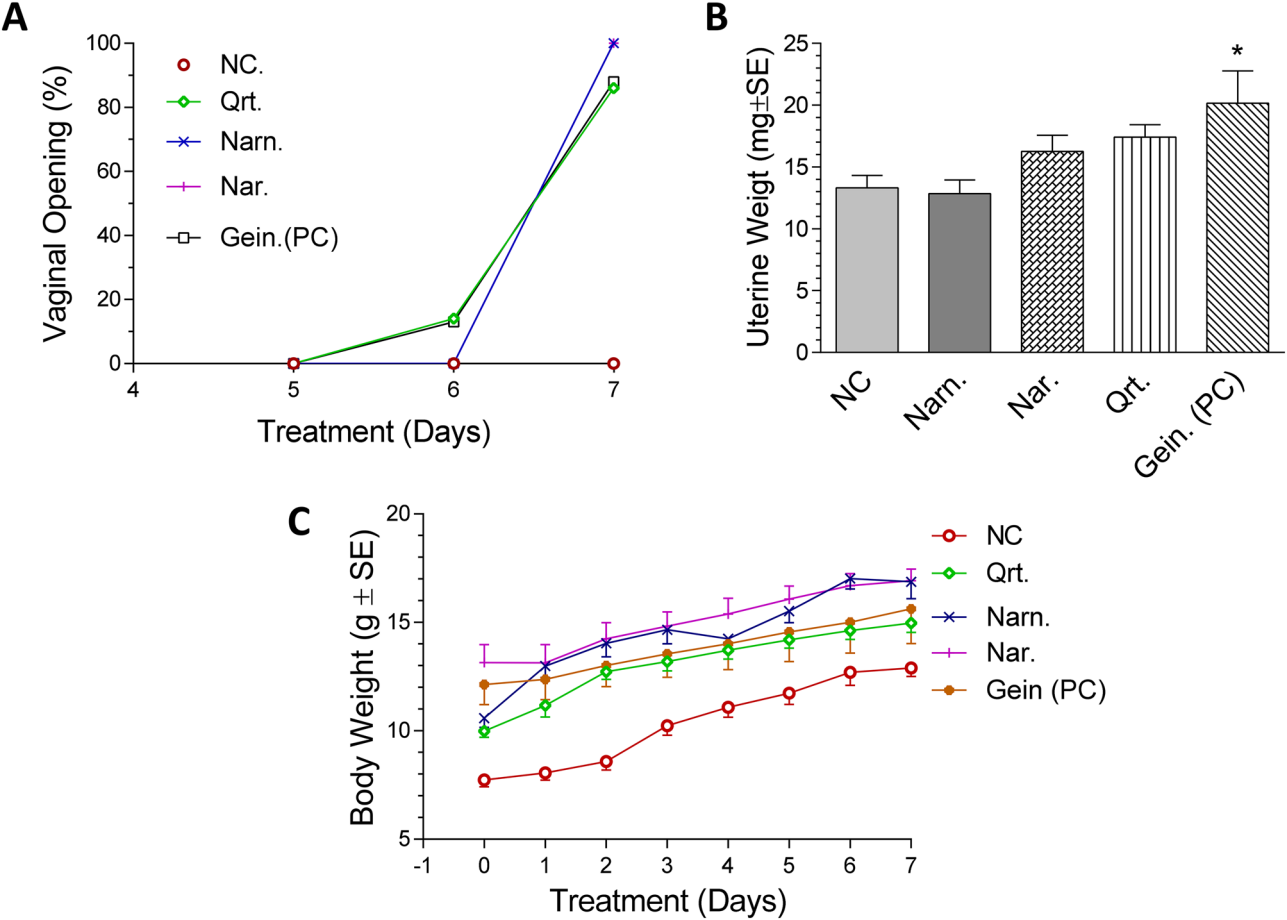
Estrogenic activity of quercetin, naringenin and naringin. (**A**) Vaginal opening (%) for the in vivo estrogenic activity groups of immature female mice. (**B**) Bar chart of average uterine weights (mg ± S.E.) of the in vivo estrogenic activity groups of female immature mice. (**C**) Body weight changes (g ± S.E.) of the in vivo estrogenic activity groups of immature female mice. *NC* negative control group receiving olive oil s.c., *Qrt* quercetin, *Narn* naringenin, *Nar* naringin, *Gein (PC)* genistein positive control phytoestrogen group. *Significant at *p* < 0.05 compared to negative control (NC) using One Way ANOVA followed by Dunnett’s Multiple Comparison test (n = 7–8). For vaginal opening, Log Rank test “Chi Square test” was used (n = 7–8).

As shown in Fig. 3A, it was found that vaginal opening started at day 6 for quercetin whereas for naringenin and naringin vaginal opening wasn’t evident before the 7th day just before termination. All flavonoids treated groups did not record any significant uterotrophic activity at the selected dose manifested in increased uterine weights, as compared to geinstein. The average uterine weights of the flavonoid treated groups as in Fig. 3B were similar to the negative control receiving only olive oil (13.0 mg ± 2.6) where quercetin, naringenin and naringin treated groups recorded average uterine weights of 13.0 ± 3.0, 16.0 ± 3.7 and 17.0 ± 2.6 mg; respectively, versus genistein positive control which recorded significant increase in uterine weight (20.0 ± 7.5 mg). Concerning the vaginal cornification, all treated groups recorded almost no cornification suggesting the absence of estrogenic activity in comparison to genistein positive control with mild vaginal cornification. It is noteworthy that body weights showed no significant differences compared to the negative control group (Fig. 3C).

### In vivo anti-estrogenic activity assay

**Figure 4:**
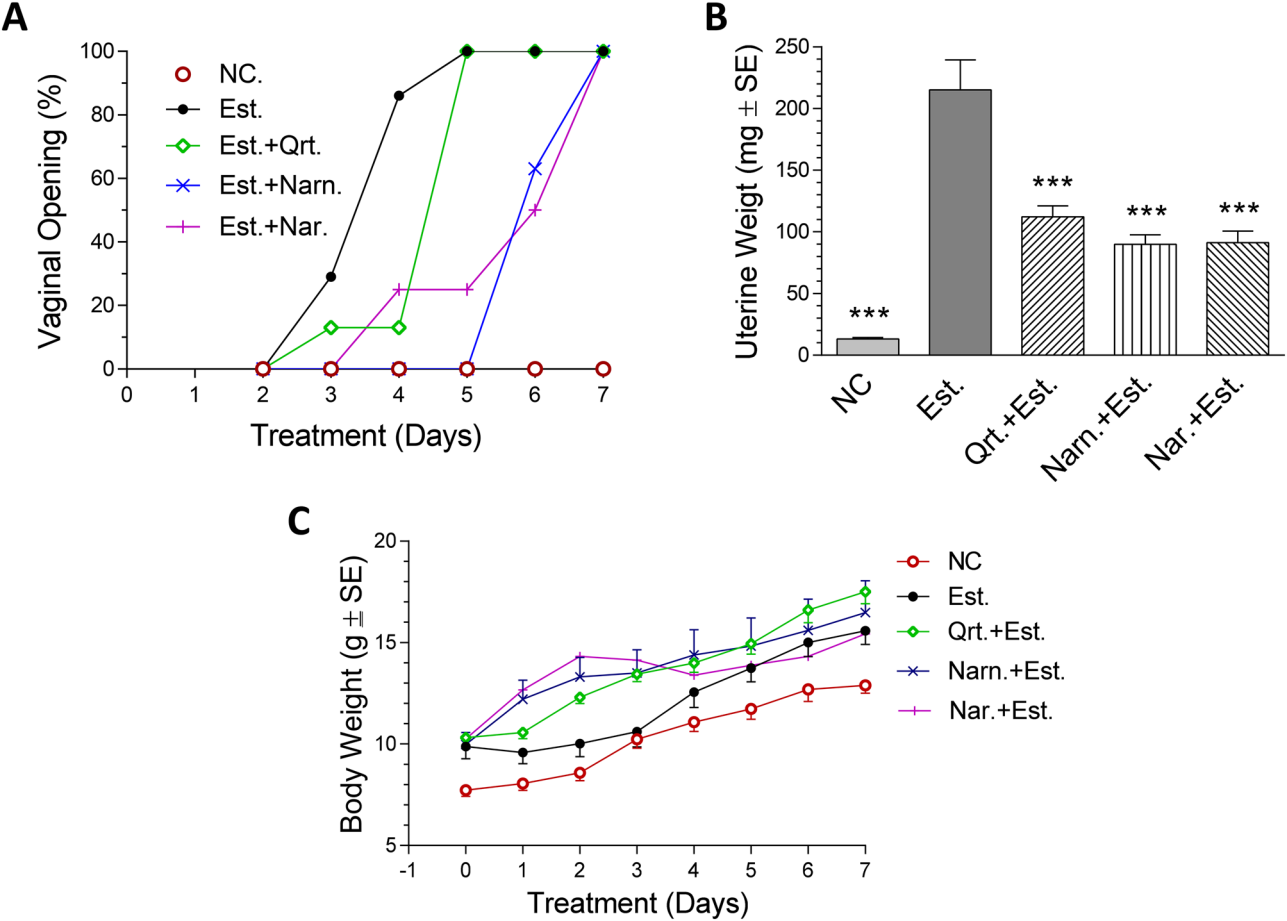
Anti-estrogenic activity of quercetin, naringenin and naringin. (**A**) Vaginal opening (%) for the in vivo anti-estrogenic activity groups of immature female mice. (**B**) Bar chart of average uterine weights (mg ± S.E.) of the in vivo anti-estrogenic activity groups of female immature mice. (**C**) Body weight changes (g ± S.E.) of the in vivo anti-estrogenic activity groups of immature female mice. *NC* negative control group receiving olive oil s.c., *Qrt* quercetin, *Narn* naringenin; *Nar* naringin, *Est.* estradiol positive control group. ***Significant at *p* < 0.0001 compared to Est. using One Way ANOVA followed by Dunnett’s Multiple Comparison test (n = 7–8). For vaginal opening, Log Rank test “Chi Square test” was used (n = 7–8).

All treated groups recorded high rates of vaginal openings as in Fig. 4A due to the estrogen like effect of estradiol achieved with flavonoid treatment. The positive estradiol control group recorded the highest rate of vaginal opening starting from day 2 reaching 100% by day 5 of treatment. The anti-estrogenic activity of the flavonoid treated groups caused a delay in the vaginal openings of immature female mice. This was exemplified in naringenin with estradiol treated group (the delay was up to day 5 and reached 100% at day 7 of treatment), followed by naringin with estradiol treated group (the delay was up to day 3 and reached 100% at day 7 of treatment) then quercetin with estradiol treated group (the delay was up to day 2 and reached 100% at day 4 of treatment) although this delay was statistically insignificant.

As shown in Fig. 4B, Naringenin and naringin flavonoids showed the most potent in vivo anti-estrogenic activity reaching 58 and 57.7% reductions in uterine weights, respectively, followed by quercetin that caused 48% reduction in uterine weight at the given dose, as compared to the positive group receiving estradiol alone (215.0 ± 64.4 mg). Notably, estradiol-induced vaginal cornification was not fully reversed by quercetin, naringenin and naringin treatment.

It is worth mentioning that no mortalities were reported in any of the treated groups. Livers also demonstrated normal morphology. No significant difference in body weight gain (Fig. 4C) in treated groups (15.0–17.0 g) compared to negative control and estradiol groups (13.0 ± 1.04 g and 15.6 ± 1.8 gm, respectively).

### Aromatase inhibition assay

Quercetin, naringenin and naringin showed inhibitory potential against aromatase enzyme in vitro, where their IC_50_ values were comparable to or even lower than that of the standard drug, ketoconazole (Table 3).

### In vivo anticancer effect and aromatase levels in tumors

**Figure 5:**
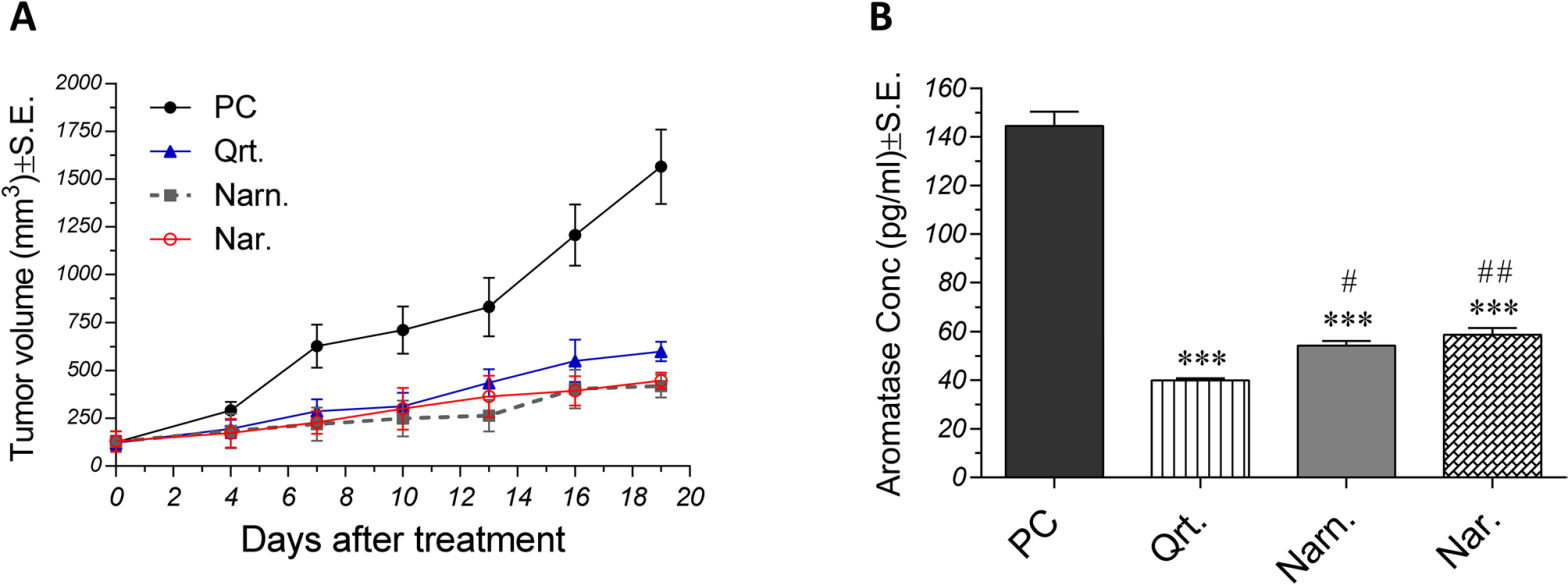
Effect of quercetin, naringenin and naringin on tumor volumes and aromatase levels in solid tumors. (**A**) Effect of flavonoids on tumor volumes (means ± S.E.) of Ehrlich Ascites Carcinoma (EAC)-bearing mice. Treatment was started 5 days after inoculation of EAC cells. Tumor volumes were measured at 4, 7, 10, 13, 16, and 19 days after starting treatment. (**B**) Effect of flavonoids on aromatase levels (means ± S.E.) in solid tumors of EAC-bearing mice determined using ELISA. Data were analyzed using One Way ANOVA followed by Tukey–Kramer post hoc test (n = 10). ***Significant at *p* < 0.0001 compared to control, ^#^Significant at *p* < 0.05 compared to quercetin, and ^##^Significant at *p* < 0.01 compared to quercetin. *PC* positive control, *Qrt* quercetin, *Narn* naringenin, *Nar* naringin.

As shown in Fig. 5A, tumor volumes were significantly decreased in treated groups relative to positive control, especially at later time points. At the 19th day after treatment (just before sacrification), quercetin-treated group showed a 61.71% decrease in tumor volume, as compared to positive control. Moreover, naringenin and naringin treated groups showed 73.26 and 71.47% lower tumor volumes compared to the control untreated group, respectively. No significant difference between treatments was observed. Likewise, aromatase levels in tumors belonging to treated groups were significantly lower than that of the control group. Moreover, as shown in Fig. 5B, aromatase levels in quercetin, naringenin and naringin groups were 72.35, 62.43, and 59.36% lower than that observed in the positive control group, respectively. Interestingly, quercetin caused 26.4 and 32% reductions in aromatase levels, as compared to naringenin and naringin, respectively.

## Discussion

Despite the exerted efforts for early diagnosis and treatment of breast cancer, the incidence is still remarkably increasing. Following surgical intervention, more than 20% of patients suffer from recurrence at a certain point of their lives. Most breast tumors (~ 80%) highly express ERs promoting cancer cell proliferation. These estrogen-dependent tumors are affected by numerous genetic and physiological factors upon which different risk groups are identified where high-risk ones are recommended to be placed on a prophylactic regimen of ER modulators at their menopause to decrease the risk/chances of developing the disease^34^.

In postmenopausal breast cancer patients, primary/neoadjuvant endocrine-based therapy is usually prompted for 4–8 months before surgery or until maximum response and resumed post-operatively. Aromatase inhibitors are, however, more effective than endocrine-based therapy in reducing tumor size, therefore leading to a less extensive surgery^35^. Synthetic aromatase inhibitors of cyano-imidazole or triazole structure such as letrozole and anastrozole are currently marketed for this purpose. Recently, research has identified some plant flavonoids such as chrysin and procyandin B to possess aromatase inhibitory activity, however, due to their moderate activity, they have not been marketed yet. In an attempt to search for more potent safe interventions, we prompted to explore the Egyptian herbal flora for plants rich in flavonoids to evaluate their anti-estrogenic and anti-aromatase activities offering prophylactic/therapeutic advantage to both premenopausal and postmenopausal women.

Citrus fruits as well as their peels are excellent sources of flavonoids such as hesperetin, naringenin, quercetin, and diosmin among others which are known to possess different biological activities and numerous health benefits^36^. On the other hand, flavonoids were found to interact with different genes and key enzymes involved in cell proliferation, cell cycle, apoptosis, angiogenesis and multidrug resistance and hence, they were studied for their chemo-preventive as well as chemotherapeutic potential in cancer^37,38^.

**Figure 6:**
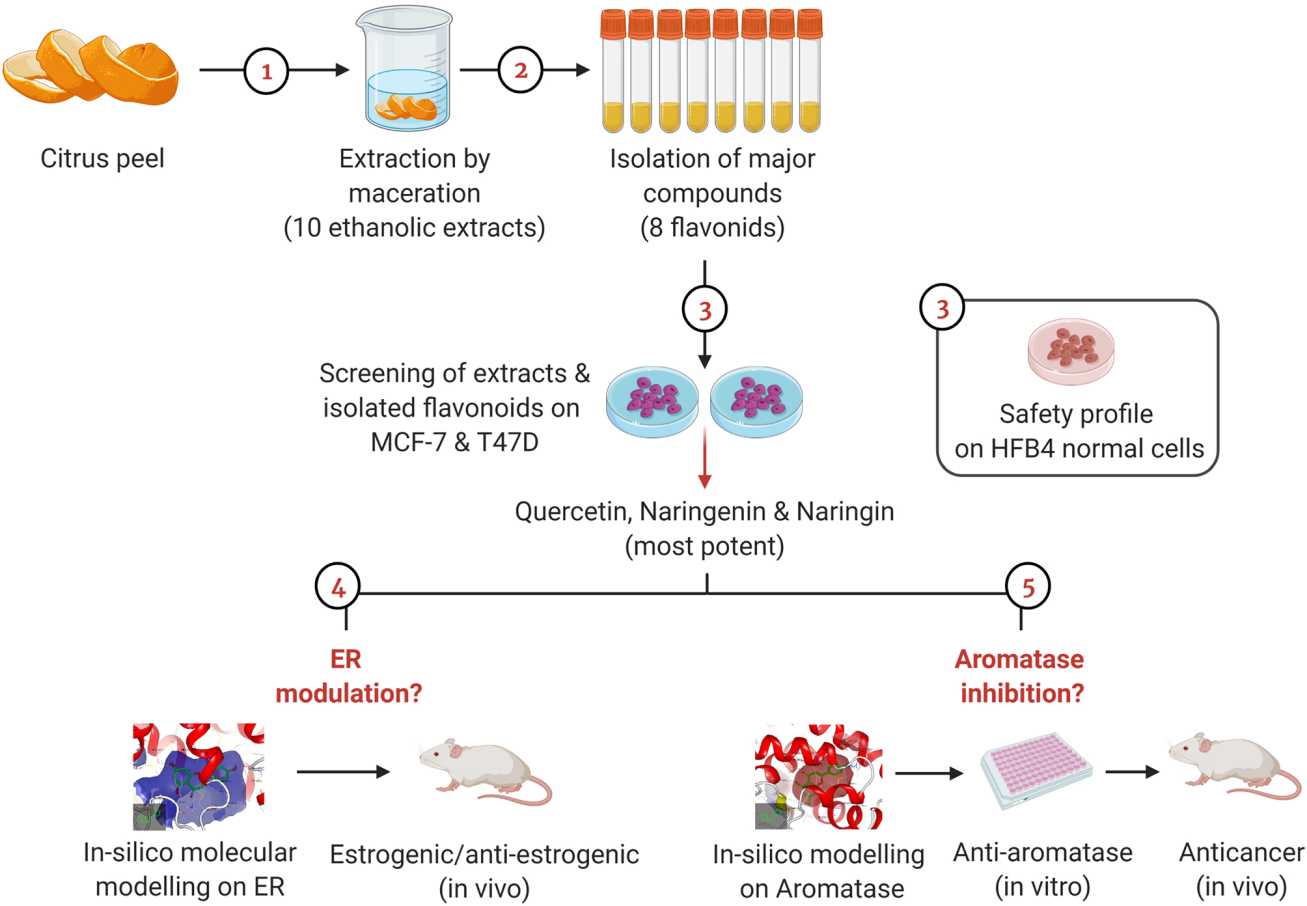
Schematic diagram outlining the sequential steps adopted in the present study. Extraction by maceration was performed for different varieties of different citrus peels. The ethanolic extracts alongside eight isolated flavonoids were screened against estrogen-dependent breast cancer cell lines (MCF-7 and T47D) as well as HFB4 normal cells for evaluating the safety profile. Naringenin, naringin and quercetin were found to be the most potent and were thus selected for further assays. The experiments performed had two arms which aimed at investigating estrogen receptor (ER) modulation and aromatase inhibition potential of the three most potent compounds. To achieve this aim, molecular docking against ER was performed followed by in vivo estrogenic and anti-estrogenic assays for the isolates. Moreover, molecular docking against aromatase enzyme was performed for the three compounds followed by in vitro aromatase inhibition. Then, investigation of the in vivo anticancer activity was executed along with estimating aromatase levels in solid tumors. This figure was created using BioRender software (www.biorender.com).

In the current study, cytotoxicity assays against estrogen-dependent breast cancer cell lines (MCF7 and T47D) were used for screening of the ethanolic extracts of the peels from ten citrus species and their isolated flavonoids. Three isolates, namely, quercetin, naringenin and naringin showed the highest potency on the aforementioned cell lines in absence of noticeable cytotoxicity on the normal melanocyte cell line, HFB4, suggesting their safety profile. Accordingly, these three flavonoids were enrolled in further assays to dissect the exact mechanism by which cytotoxicity was achieved. And since these cell lines are estrogen-dependent, we attempted to verify whether this was due to ER modulation or interfering with estrogen biosynthesis via aromatase inhibition. A summary of the approach adopted in the current study presented as sequential steps is provided in Fig. 6.

Previously, the mechanistic roles of citrus peel flavonoids as potential anticancer agents were discussed^3^. Citrus flavonoids viz*.* hesperitin, naringenin and nobelitin were found to induce cell cycle arrest at different phases of the cell cycle^39^. Suppression of cancer cell proliferation and induction of apoptosis were also achieved by citrus flavonoids via a caspase-dependent mechanism as well as triggered calcium influx^40^. Naringenin, a major citrus peel flavonoid was also found to suppress the upregulation of matrix metalloproteinase-9 as well as tumor necrosis factor-α which mediated the release of IL-6 and IL-8 posing a potential impact on cancer cell migration and metastasis^41^. Angiogenesis is also considered an important process for cancer cell proliferation and metastasis. Interestingly, citrus flavonoids have demonstrated an anti-angiogenic potential by inhibiting the vascular endothelial growth factor^42^. Flavonoids have also exhibited antiproliferative and anti-aromatase activities on estrogen-dependent breast cancer cell lines^43,44^.

To provide further mechanistic insights, the superimposition of the three compounds was assessed using 17-β-estradiol and androsterone structures as starting points for validating the molecular mimicry of the proposed compounds to the ER targeting ligands and aromatase enzyme, respectively. Evaluation of the superimposition of the 3D structures showed that only naringenin and quercetin had high degrees of superimposition. Further validation was conducted via receptor-based approach by conducting semi-flexible docking for multi-conformers of quercetin, naringenin and naringin, in comparison with 17-β-estradiol and androsterone, against the crystal structure of the ligand binding domain of ER and aromatase enzyme, respectively, where naringenin and quercetin exhibited hydrophobic–hydrophobic interactions along with both targets.

To validate the in-silico findings, biological evaluation of the anti-estrogenic and anti-aromatase activities were performed. In vivo estrogenic and anti-estrogenic activity of quercetin, naringenin and naringin showed significant anti-estrogenic activity based on the effect on vaginal opening and uterine weights. A relevant study previously reported that naringenin at a dose of 30 mg/rat co-treated with estradiol (0.5 µg/rat) showed a significant decrease in estrogen-induced uterine weight which is indicative of the anti-estrogenic activity exhibited by naringenin^45^. Another study conducted on both naringin and naringenin showed that they exhibited double directional estrogenic and anti-estrogenic activities at a dose of 40 and 80 mg/kg, respectively. Estrogenic activity was achieved at low stilbesterol levels however at high estrogen content, they acted as anti-estrogenic^28^. Quercetin, a phytoestrogenic compound, showed estrogenic and anti-estrogenic activities, according to the selected dose. At 10 mg/kg, quercetin had an anti-estrogenic effect on the uterine weight upon treatment with steroid, but at a higher dose (100 mg/kg), it exhibited potent estrogenic effect stimulating carcinogenesis^46^. Moreover, the study of Lacopetta et al*.*^47^ provided further insights into the cytotoxic mechanism of action of quercetin and its analogs where an inhibition of human topoisomerases types I and II along with scavenging potential for reactive oxygen species were reported.

On the molecular level and active binding sites for the aromatase enzyme, a previous study reported that the presence of a hydroxy group at carbon no 7 on the flavone nucleus was found to interact with Ser478 and hence it is important for binding to the aromatase enzyme. Moreover, the substitutions at ring B usually led to a reduction in the activity. This reduction was due to negative interactions with the hydrophobic active site residues^48^. Furthermore, previous studies also reported a potent anti-aromatase activity for flavonoids; by substituting ring-B of flavanones with 7- methoxy group. Hence, it was concluded that hydroxylation at C-3 and/or C-4 promoted the anti-aromatase activity^8^.

In order to explore whether quercetin, naringenin, and naringin were capable of influencing aromatase levels in solid tumors, an in vivo animal model of EAC solid tumor was used and aromatase levels were determined in tumor homogenates of control and treated groups. The three compounds were found to significantly decrease tumor volumes as well as aromatase levels. This goes in accordance with previous studies showing that different flavonoids influenced aromatase activity which was reflected on alterations in aromatase expression levels^49,50^.

In view of the anti-estrogenic and aromatase inhibitory activities of quercetin, naringenin and naringin isolated from citrus peel extracts, this study proposes exploiting citrus peels in isolating natural phytochemicals for possible incorporation in chemotherapy regimens tailored for premenopausal and postmenopausal breast cancer patients based on the efficacy and safety profiles they possess. These findings also suggest their use for prophylactic purposes in women at risk for developing the disease. Future clinical studies, however, are needed to guarantee the promising effects of these flavonoids.

## Supplementary Information

Supplementary Information.
