# Supplementary material for: Anti-estrogenic and anti-aromatase activities of citrus peels major compounds in breast cancer
Source: Sci Rep. 2021 Mar 29;11:7121. doi: 10.1038/s41598-021-86599-z (PMC8007834; doi:10.1038/s41598-021-86599-z)
Supplement: Supplementary file 1 — Supplementary Information. [file 41598_2021_86599_MOESM1_ESM.pdf]

Anti-estrogenic and anti-aromatase activities of Citrus peels major compounds in breast cancer

Dina M. El-Kersh[1], Shahira M. Ezzat[2,3*], Maha M. Salama[1,2], Engy Mahrous[2], Yasmeen M. Attia[4], Mahmoud Salama Ahmed[5], Mohey M. Elmazar[4]

[1]Department of Pharmacognosy, Faculty of Pharmacy, The British University in Egypt, El Sherouk City, Suez Desert Road, Cairo 11837, Egypt.
[2]Department of Pharmacognosy, Faculty of Pharmacy, Cairo University, Kasr El-Aini Street, Cairo 11562, Egypt.
[3]Department of Pharmacognosy, Faculty of Pharmacy, October University for Modern Sciences and Arts (MSA), 6th October 12611, Egypt.
[4]Department of Pharmacology, Faculty of Pharmacy, The British University in Egypt, El Sherouk City, Suez Desert Road, Cairo 11837, Egypt.
[5]Department of Internal Medicine, Division of Cardiology, The University of Texas Southwestern Medical Center, Dallas, TX, USA

*Corresponding author: Maha M. Salama, Department of Pharmacognosy, Faculty of Pharmacy, Cairo University, Kasr El-Ainy St., Cairo11562, Egypt. Tel.: +201008325473; E-mail: maha.salama@pharma.cu.edu.eg

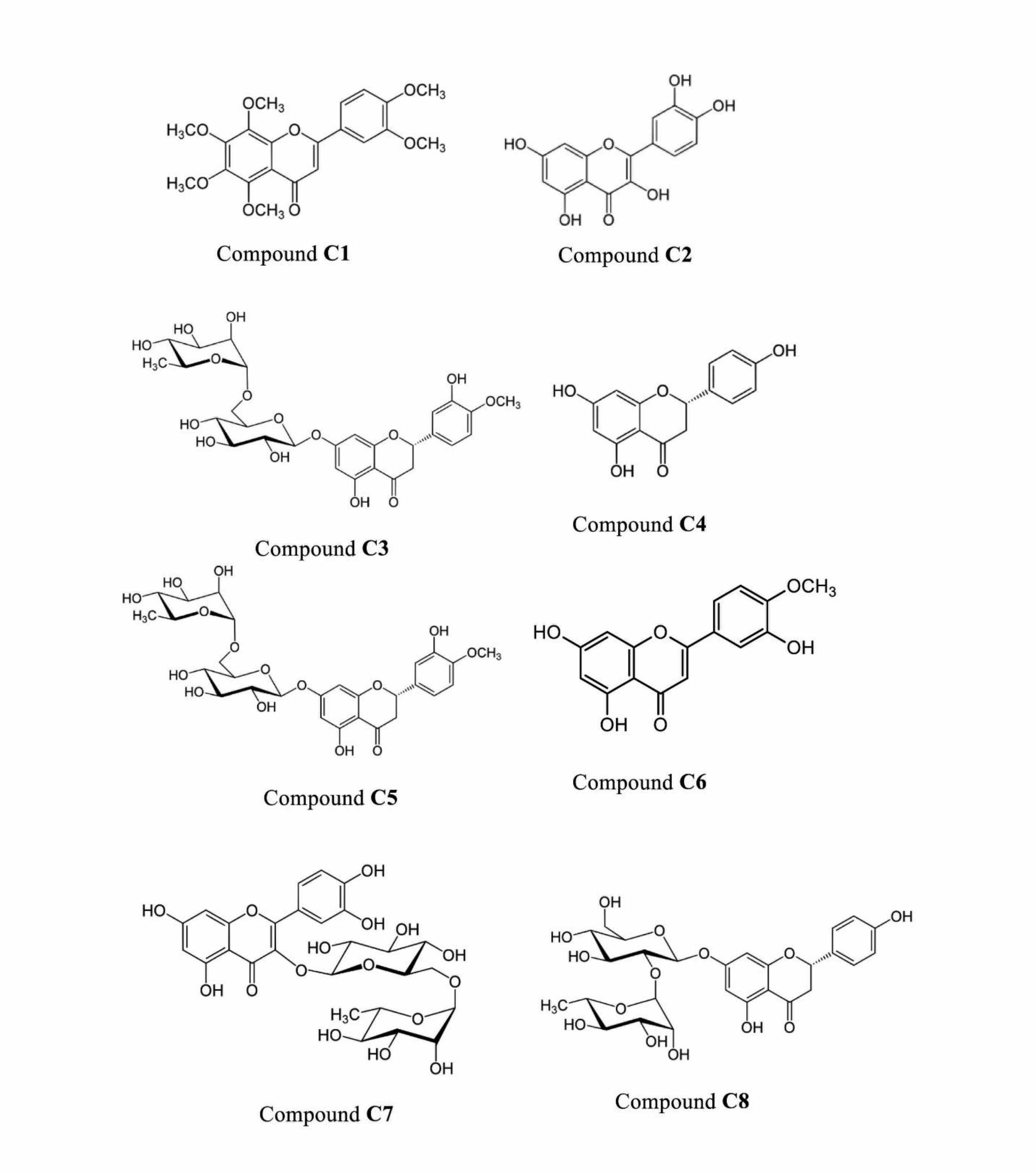

Supp. Figure 1: Chemical structures of the isolated compounds. C1: nobiletin; C2: quercetin; C3: diosmin; C4: naringenin; C5: hesperidin; C6: hesperitin, C7: rutin and C8: naringin.
